# Histological observations on aural fibrosarcoma in a Holstein cow

**DOI:** 10.1002/vms3.1126

**Published:** 2023-03-21

**Authors:** Sara Shokrpoor, Morteza Gorjidooz, Peyman Azizi, Seyed Mehdi Ghamsari

**Affiliations:** ^1^ Faculty of Veterinary Medicine Department of Pathology University of Tehran Tehran Iran; ^2^ Faculty of Veterinary Medicine Garmsar Branch Department of Clinical Sciences Islamic Azad University Garmsar Iran; ^3^ Faculty of Veterinary Medicine Department of Surgery and Radiology University of Tehran Tehran Iran

**Keywords:** fibrosarcoma, histopathology, Holstein cow, immunohistochemistry, tumour

## Abstract

Fibrosarcomas occur as a mesenchymal tumour of malignant fibroblasts in a collagen background and are usually found in the female genital organs and rarely involve the skin. A 5‐year‐old female Holstein cow with a raised mass at the base of right ear was referred. On gross examination, the mass was approximately 13.00 × 10.00 × 7.00 cm in size. Finally, complete surgical removal was selected. The mass was encapsulated and the dermis was expanded by spindle‐shaped to polygonal neoplastic cells. These cells were arranged in interwoven pattern. Mitotic figures were infrequent. Masson's trichrome demonstrated the positive and blue staining of collagen. Immunohistochemically, the sections were uniformly positive for Vimentin and negative for Desmin, SMA and GFAP. A well‐differentiated fibrosarcoma was diagnosed based on histopathological features. Surgical excision is the treatment of choice for this neoplasm. In the present case, surgery was also performed successfully and no new growth of the mass was observed 4 months following the surgical procedures. To our knowledge, this is the first report of well‐differentiated fibrosarcoma in a Holstein cow.

## Graphical abstract

This report describes the aural fibrosarcoma in a 5‐year‐old female Holstein cow. In the text of the study, macroscopical, microscopical and immunohistochemical findings are described.

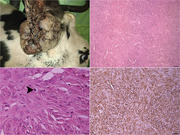

## 1 INTRODUCTION

Fibrosarcomas are malignant connective tissue tumours with immature proliferating fibroblasts or undifferentiated anaplastic spindle cells arranged in interwoven pattern (Hendrick, 2017). These tumours have been reported in cats (Strong et al., 2016), dogs (de Paula et al., 2021) and horses (Bass et al., 2017). There are only rare reports of bovine vaginal (Avci et al., 2010; Mushap, 2016), penile (Hesaraki et al., 2010), mandibular (Braun et al., 2001) and mammary gland fibrosarcoma (with metastases in local lymph nodes) (Orr, 1984) in the literature. Bovine cutaneous fibrosarcoma is an unusual tumour. To our knowledge, there has been no report of this tumour in the Holstein cow. This paper is about the macroscopic, surgical and histopathological findings of aural well‐differentiated fibrosarcoma in a Holstein cow.

## 2 CASE DESCRIPTION

**FIGURE 1:**
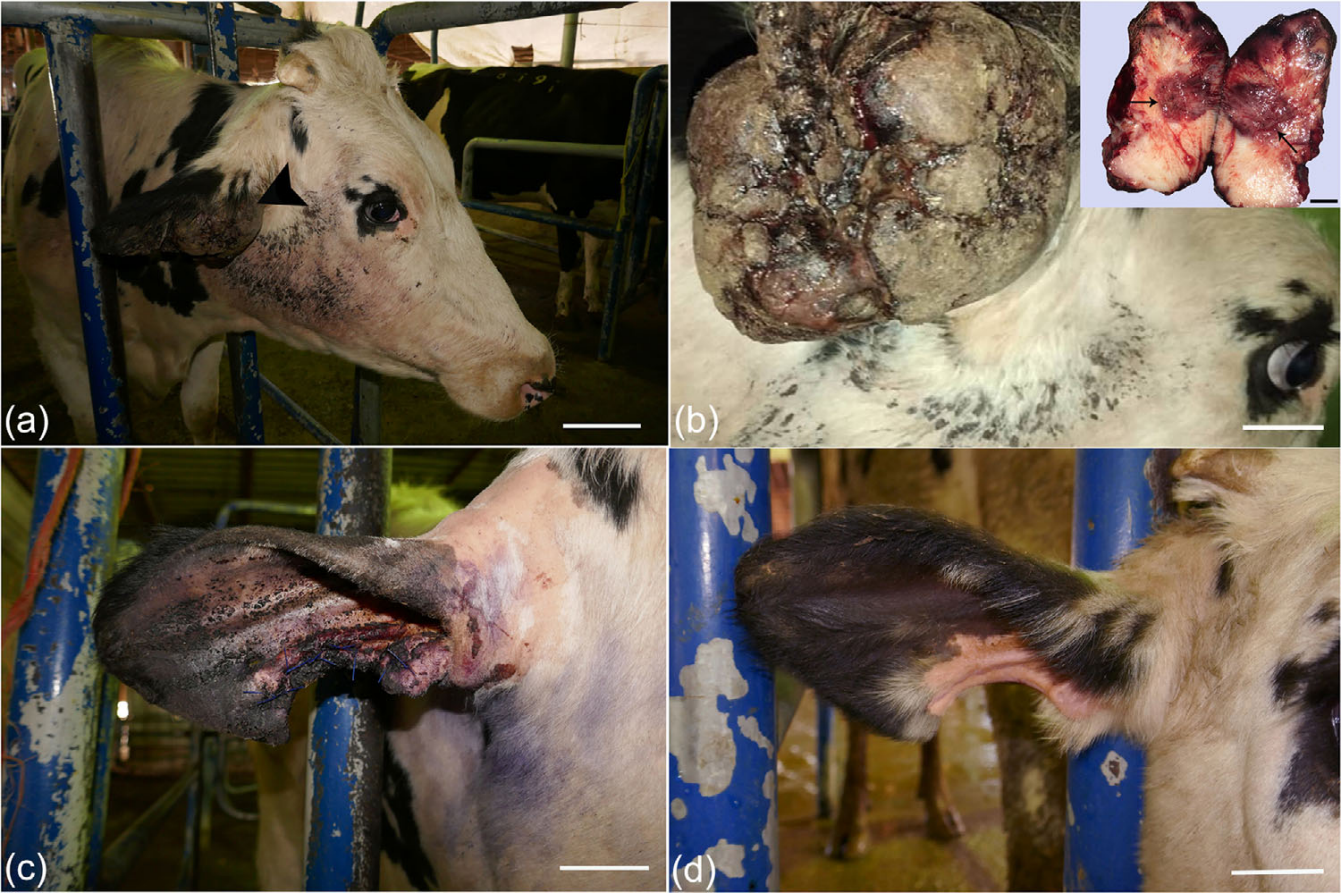
(a, b) A raised mass at the base of right ear (arrowhead), central necrosis of the tumour (arrows). (c) Holstein cow's ear after the complete surgical removal. (d) Surgical wound healing (scale bars = 5.00 cm).

**FIGURE 2:**
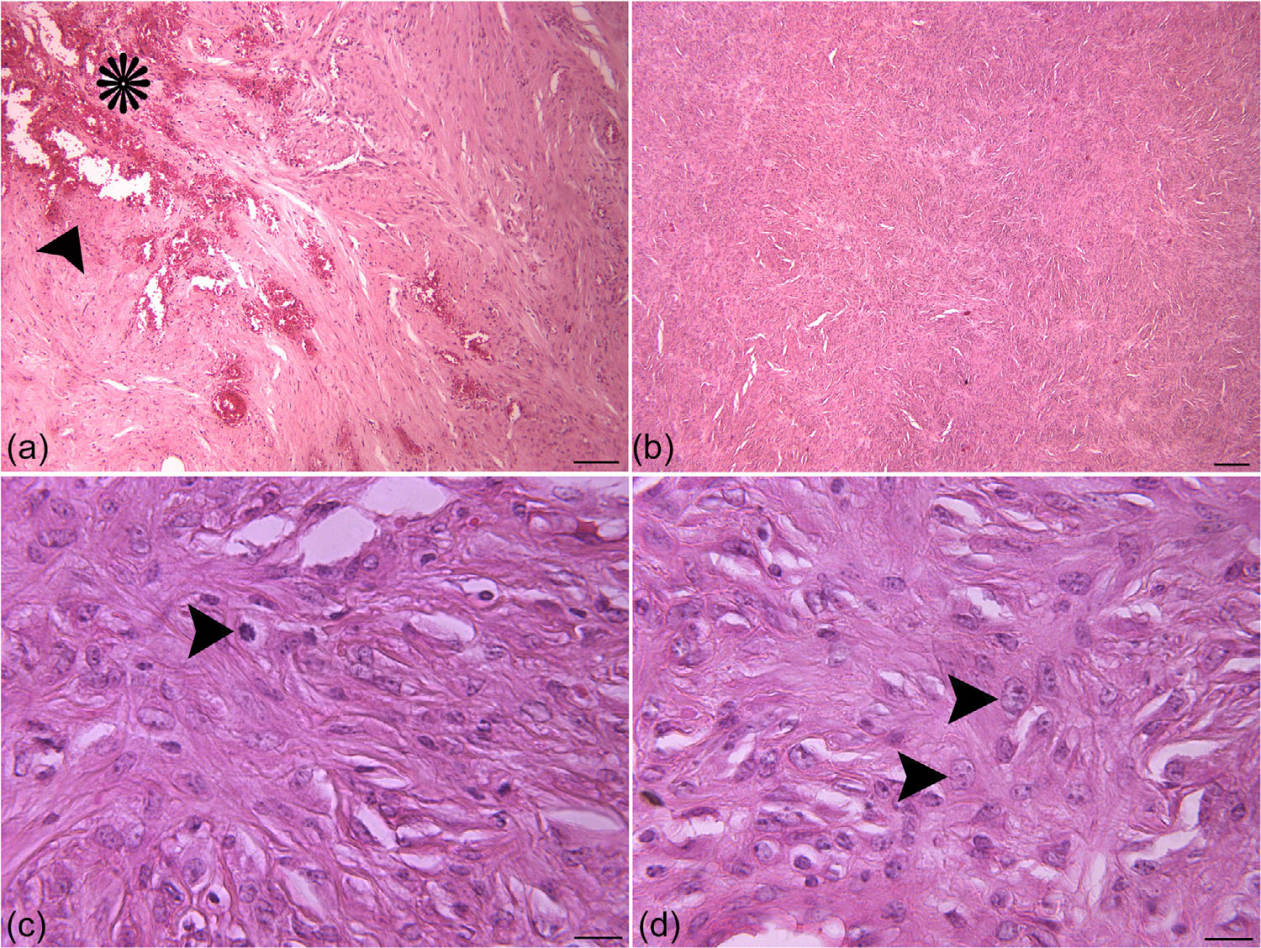
(a) Note the haemorrhage (*) and necrosis (arrowhead) (H&E, scale bar = 100 µm). (b) Neoplastic cells in interwoven pattern (H&E, scale bar = 200 µm). (c) Mitotic figure (arrowhead) (H&E, scale bar = 20.00 µm). (d) Bizarre cells (arrowheads) (H&E, scale bar = 20.00 µm).

**FIGURE 3:**
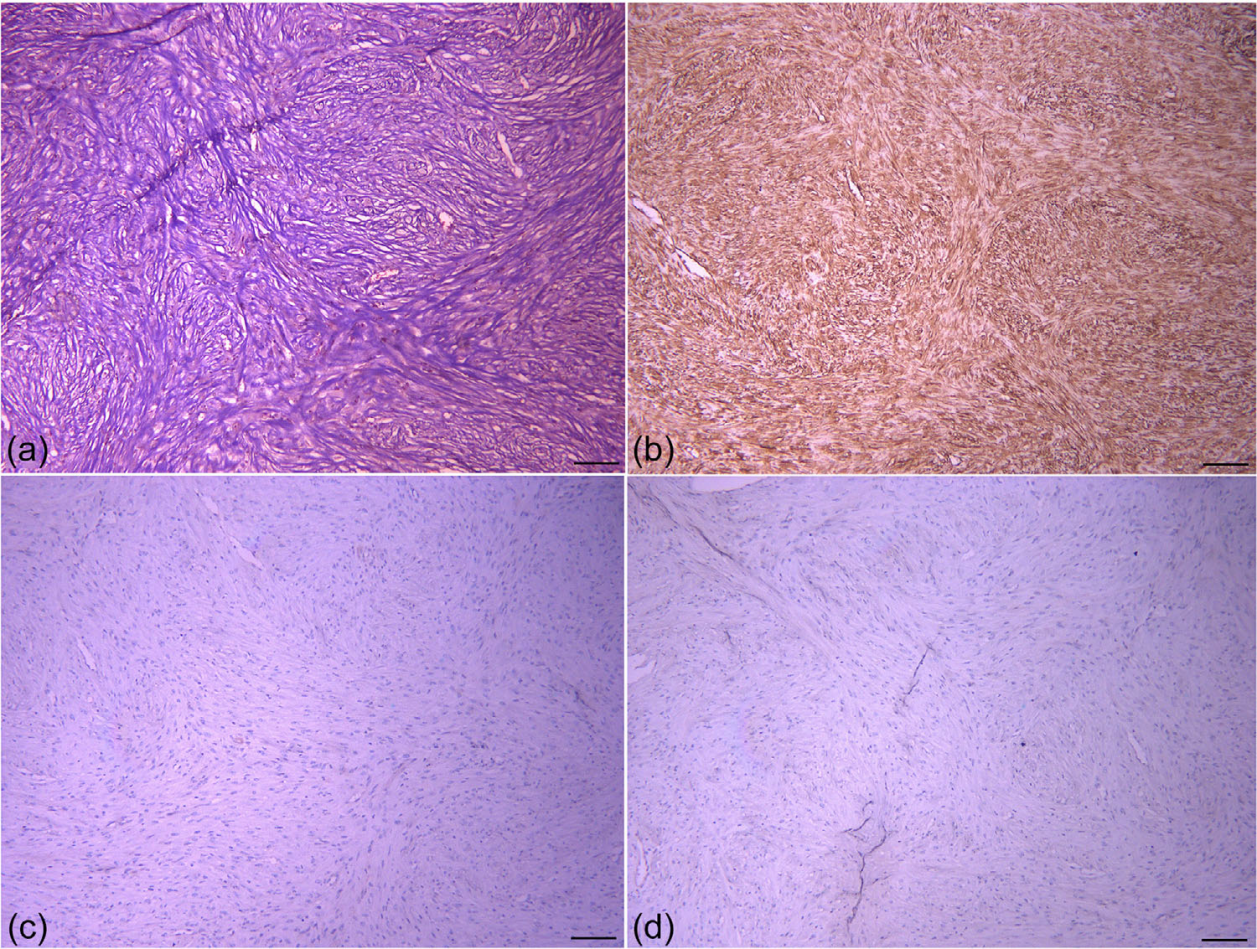
(a) Masson's trichrome positive of collagenous fibres (Masson's trichrome stain, Scale bar = 100 µm). (b) Strong and diffuse reactivity for Vimentin (IHC, scale bar = 100 µm). (c) No immunoreactivity to Desmin and (d) SMA in the neoplastic cells (IHC, scale bar = 100 µm).

**FIGURE 4:**
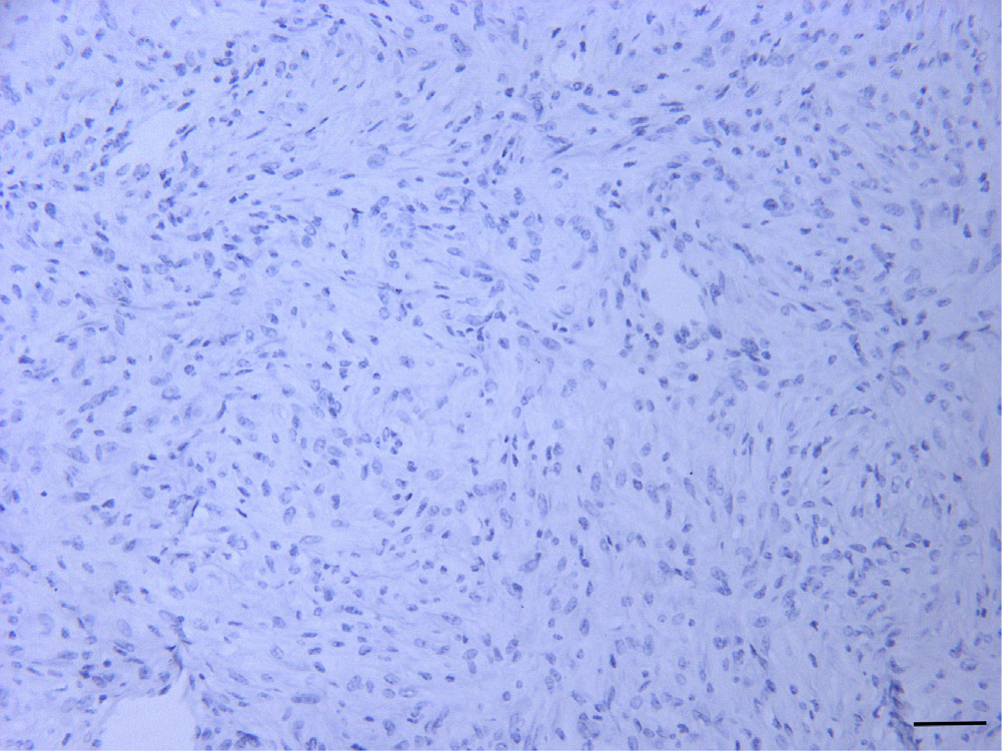
No immunoreactivity to GFAP in the neoplastic cells (IHC, scale bar = 50 µm).

In December 2021, a 5‐year‐old female Holstein cow with a raised mass at the base of right ear was referred (Figure 1a and b). Based on owner information, within the previous 1‐month period the mass had become evident and grew larger. On physical examination, the vital signs as pulse rate, respiratory rate and rectal temperature were normal. Further clinical examination revealed no other physical abnormalities. On gross examination, the lobulated mass was approximately 13.00 × 10.00 × 7.00 cm in size. Finally, complete surgical removal was selected, and surgical resection was carried out under local anaesthesia with lidocaine hydrochloride 2.00% and xylazine. The mass was removed for histopathological evaluation, and the incision was sutured by horizontal mattress sutures (Figure 1c). On section, the mass was well circumscribed, ulcerated, firm and white to yellow. Tissue samples of the mass were fixed in 10.00% neutral buffered formalin, routinely processed, dehydrated, embedded in paraffin wax, sectioned at 5.00 µm in thickness (Rotary Microtome RM2 145; Leica, Wetzlar, Germany) and stained with Haematoxylin and Eosin. Sections were examined using a light microscope (E600; Nikon, Tokyo, Japan) and representative images were taken. The formalin‐fixed mass sections were also stained with Masson's trichrome (Luna, 1968). For this reason, the sections (5 µm) were deparaffinised, rehydrated and wash. These were stained in Weigert's iron haematoxylin for 10 min, rinsed and washed. The slides were stained in Biebrich scarlet‐acid fuchsine solution for 12 min, washed and differentiated in phosphomolybdic‐phosphotungstic acid solution. The sections were transferred to aniline blue solution for 7 min, rinsed and differentiated in 1% acetic acid solution. Then the slides were washed, dehydrated and mounted. For immunohistochemistry, the avidin biotin‐ peroxidase complex (ABC) method was used with primary antibodies to Vimentin (V9, mouse monoclonal, dilution: 1/50, Dako, Denmark) (Luna, 1968), SMA (1A4, mouse monoclonal, dilution: 1/50, Dako, Denmark), GFAP (GF5, mouse monoclonal, dilution:1/100, Dako, Denmark) (Kelley et al., 1994) and Desmin (D33, mouse monoclonal, dilution: 1/50, Dako, Denmark) (Vascellari et al., 2006). Serial sections (5 µm) were placed on poly‐L‐lysine (Sigma) coated slides. Following incubation for 12 h at 37°C, sections were deparaffinised, and rehydrated. Endogenous peroxidase activity was blocked by incubation with 3% H_2_O_2_ /methanol. The sections were incubated with the primary antibodies overnight at 4°C, washed with phosphate‐buffered saline (PBS) and were incubated with the biotinylated secondary antibody for 30 min at room temperature. The sections were rinsed with PBS and reacted with streptavidin‐peroxidase conjugate for 30 min. After the slides were washed, treated with the substrate chromogen (3, 3’‐diaminobenzidine, DAKO), counterstained with Mayer's haematoxylin, dehydrated, and mounted. Antibacterial therapy was applied with a pen & strep (0/10 mg/kg, IM, SID for 5 days) and flunixin meglumine 5.00% (2/20 mg/kg, IM, SID for 3 days). In histopathological investigations, haemorrhage, necrosis and peripheral aggregates of lymphocytes were observed (Figure 2a). The mass was encapsulated and the dermis and subcutaneous area were expanded by spindle‐shaped to polygonal neoplastic cells. These cells were arranged in interwoven pattern (Figure 2b) and contained a single ovoid to polygonal nucleus and a moderate amount of eosinophilic cytoplasm. Nucleoli were often prominent. Mitotic figures were infrequent (Figure 2c). Some neoplastic cells had cellular and nuclear pleomorphism (Figure 2d). Masson's trichrome demonstrated the positive and blue staining of collagen (Figure 3a). Immunohistochemically, the sections were uniformly positive for Vimentin (Figure 3b). They stained negative for Desmin (Figure 3c), SMA (Figure 3d) and GFAP (Figure 4). A well‐differentiated fibrosarcoma was diagnosed based on histopathological, histochemical and immunohistochemical features. Based on owner information, no new growth of the mass was observed 4 months following the surgical procedures (Figure 1d).

## 3 DISCUSSION

Fibrosarcomas are common tumours in domestic animal species, and can range from well differentiated to more aggressive, but metastasis is uncommon (Hendrick, 2017). Well‐differentiated fibrosarcoma is composed of spindle cells, collagen and rare mitotic figures in the herring bone pattern (Hendrick, 2017; Van den Top et al., 2008). Deltapapillomavirus (BPV‐1 and BPV‐2) and Epsilonpapillomavirus (BPV‐5 and BPV‐8) have been isolated from fibroepithelial tumours (fibropapillomas) in cattle (Silva et al., 2013). Fibropapillomas are the most common bovine cutaneous tumours. Fibrosarcomas, unlike fibropapillomas, are rare (Silva et al., 2013). In cattle, Fibrosarcomas occur as a mesenchymal tumour of malignant fibroblasts in a collagen background and are usually found in the female genital organs (Avci et al., 2010). To our knowledge, cutaneous fibrosarcoma has not been reported in Holstein cow. Similar to this case, mostly fibrosarcomas develop in adult animals (Hendrick, 2017). There is significant overlap between fibrosarcomas and leiomyosarcomas in histopathological findings. Microscopically, unlike fibrosarcomas, leiomyosarcomas have elongate nuclei, with blunt rounded ends and eosinophilic cytoplasm. However, histochemical and immunohistochemical identification are essential for an accurate histopathological diagnosis. In this case, on Masson trichrome staining, spindle cells with red cytoplasmic stain were not found among the blue collagen bundles. The mass was also stained negative for Desmin and SMA. So, these results revealed that this tumour could not originate from muscle cells. Unlike this case, positive IHC reactivity for smooth muscle actin and Desmin is supportive of a leiomyosarcoma (Hendrick, 2017; Roccabianca et al., 2020). The aural mass was not schwannoma because of its negative reactivity to GFAP (Hesaraki et al., 2010). This case is a fibrosarcoma with a reaction only to Vimentin. Surgical excision is the treatment of choice for well‐differentiated fibrosarcoma (Hendrick, 2017). Deltapapillomavirus (BPV‐1 and BPV‐2), Epsilonpapillomavirus (BPV‐5 and BPV‐8) and genetic influences are included as potential factors for fibropapilloma (Silva et al., 2013). E5, the major BPV oncoprotein, is expressed in the basal layer of the epidermis and in dermal fibroblasts (McCance, 2002), during the early and late stages of viral carcinogenesis, suggesting that E5 is causally involved in the development of the bovine cutaneous tumours (Silva et al., 2013). However, we were unable to identify an obvious etiological agent, and the cause of the fibrosarcoma in this Holstein cow remains unknown.

## 4 CONCLUSION

The present study revealed that diagnosis of fibrosarcoma in a quite rare localisation by the routine histopathological staining method (HE) may be difficult. So, concurrent evaluation of both histopathological and IHC features is required for the definitive diagnosis of this tumour.

## AUTHOR CONTRIBUTIONS

Sara Shokrpoor: validation; writing‐original draft. Morteza Gorjidooz: investigation. Peyman Azizi: investigation.

## CONFLICT OF INTEREST STATEMENT

The authors declare that there is no conflict of interest regarding the publication of this article.

## FUNDING INFORMATION

This research received no specific grant from any funding agency.

### ETHICS STATEMENT

The authors confirm that the ethical policies of the journal, as noted on the journal's author guidelines page, have been adhered to and the appropriate ethical review committee approval has been received. This study was performed based on guideline for the care and use of laboratory animals in Iran. https://doi.org/10.1038/s41684‐021‐00871‐3

### PEER REVIEW

The peer review history for this article is available at https://publons.com/publon/10.1002/vms3.1126.

## Data Availability

Data sharing not applicable to this article as no datasets were generated or analysed during the current study.
